# Objective Effects and Patient Preferences for Ambulatory Oxygen in Fibrotic Interstitial Lung Disease With Isolated Exertional Hypoxaemia: A Placebo‐Controlled 6‐Minute Walk Test Study

**DOI:** 10.1111/resp.70020

**Published:** 2025-03-11

**Authors:** Giuseppina Ciarleglio, Paolo Cameli, David Bennett, Behar Cekorja, Paola Rottoli, Elisabetta A. Renzoni, Piersante Sestini, Elena Bargagli

**Affiliations:** ^1^ Department of Medicine, Surgery and Neurosciences, Respiratory Diseases Unit University of Siena Siena Italy; ^2^ Interstitial Lung Disease Unit, Royal Brompton and Harefield Clinical Group, Guy's and St Thomas' NHS Foundation Trust, and Margaret Turner Warwick Centre for Fibrosing Lung Disease National Heart and Lung Institute, Imperial College London London UK

**Keywords:** 6‐minute walk test, ambulatory oxygen, exercise tolerance, fibrotic interstitial lung diseases, patient preferences

## Abstract

**Background and Objective:**

The available evidence on the effects of ambulatory oxygen on exercise impairment in patients with fibrotic interstitial lung diseases (F‐ILD) is of limited quality.

**Methods:**

We conducted a randomised, double‐blind, placebo‐controlled crossover trial with 32 normoxaemic F‐ILD patients, desaturating to ≤ 88% during a baseline 6‐minute walk test (6MWT) on ambient air. After determining the oxygen flow needed to prevent desaturation, patients completed two double‐blind 6MWTs with either oxygen or placebo (compressed medical air) at the same personalised flow. Objective measures included oxygen saturation, pulse rate, and distance walked. Patient‐reported outcomes, assessed via visual analogue scales, included end‐of‐test dyspnoea, fatigue, and preferences for walking with oxygen or placebo versus each other and ambient air.

**Results:**

Ambulatory oxygen, compared to placebo, prevented desaturation, reduced tachycardia, increased walking distance by 37 m (95% CI: 10–74, *p* = 0.008), and lessened dyspnoea and fatigue. The mean preference score for oxygen over placebo was 2.6 (95% CI: 1.9–3.2, *p* < 0.0005), significantly greater than equivalence. The preference score for placebo over ambient air was −1.5 (−2.4 to 0.64, *p* = 0.005), significantly lower than equivalence, while the score for oxygen over ambient air was 0.4 (−0.7 to 1.5), not significantly different from equivalence.

**Conclusions:**

Our data confirm that ambulatory oxygen provides significant benefits beyond a placebo effect; although in some patients it is associated with a negative perception that may hinder treatment acceptance. This strengthens the evidence supporting current recommendations and suggests that incorporating patient preferences recorded at the time of the 6MWT into clinical discussions can aid shared decision making regarding ambulatory oxygen.

**Trial Registration:** ClinicalTrials.gov identifier: NCT02668029

## Abstract 2

In a double‐blind, placebo‐controlled 6‐minute walk test study, patients with Fibrotic Interstitial Lung Diseases and exertional breathlessness with hypoxaemia experienced less dyspnoea and fatigue, walked farther, and preferred ambulatory oxygen at a flow rate preventing desaturation, compared to medical air at the same flow rate.

**Summary**

- In a double‐blind, placebo‐controlled 6‐minute walk test study, patients with Fibrotic Interstitial Lung Diseases and exertional breathlessness with hypoxaemia experienced less dyspnoea and fatigue, walked farther, and preferred ambulatory oxygen at a flow rate preventing desaturation, compared to medical air at the same flow rate.

## 1 Introduction

Fibrotic Interstitial Lung Diseases (F‐ILD) encompass a group of conditions with diverse pathogenesis, clinical/radiological characteristics, and longitudinal behaviour, but which share a variety of similar patho‐physiological mechanisms and clinical manifestations, ultimately leading to respiratory failure [1, 2]. Recent treatments have demonstrated the ability to slow disease progression. However, outside of pulmonary rehabilitation, no interventions are currently available to address the primary clinical issue of exercise limitation [3], which is common from the early stages of F‐ILD [4, 5, 6, 7].

Extensive in‐laboratory assessments have documented the beneficial effects of oxygen administration on many pathophysiological mechanisms underlying exercise limitation in F‐ILD [8, 9, 10, 11, 12, 13, 14, 15, 16] In the clinical setting, ambulatory oxygen has been shown to improve exercise endurance during the 6MWT (reviewed by Lin et al. [17]) and health‐related quality of life in real‐life conditions, with reduced breathlessness and improved walking ability during activity [18]. Despite these data, according to the most recent guidelines, the evidence in support of offering ambulatory oxygen in suitable patients remains low [19, 20, 21, 22]. This may be due in part to a scarcity of studies in real‐life conditions [18, 23, 24]. Furthermore, systematic reviews have reported limited quality of the available studies addressing the short‐term effects of ambulatory oxygen, relative to a lack of titration of oxygen requirements to prevent ongoing exertional desaturation or absence of double blinding [25, 26]. Therefore, there remains controversy and heterogeneity in the prescription of ambulatory oxygen for these patients [6, 27, 28].

It is crucial that the best available external clinical evidence, described as “clinically relevant research, often from the basic sciences of medicine, but especially from patient‐centred clinical research” on the efficacy of interventions, be consistently integrated in clinical decision‐making alongside individual patients' predicaments, rights, and preferences [29]. We therefore evaluated, under standardised and double‐blind conditions, both objective outcomes and patient preferences regarding breathing oxygen (at an individualised flow to prevent significant desaturation) versus medical air (placebo) at the same individualised flow, after having experienced these modalities during a 6‐minute walk test, a clinical test reproducing a common daily activity in the clinical setting, under standardised conditions.

## 2 Methods

We enrolled patients with F‐ILD in stable clinical conditions [1, 2] aged < 85 years, normoxic at rest (transcutaneous arterial oxygen saturation—SpO_2_ ≥ 92%—at rest), and experiencing a drop in SpO_2_ ≤ 88% during a baseline 6‐minute walk test (6MWT). We excluded patients unable or unwilling to provide consent, those with recent acute illness, those with walking limitations due to non‐pulmonary causes, and those who had participated in similar studies previously. Patient evaluation included blood gas analysis at rest, pulmonary function tests, and a chest x‐ray performed within 30 days of the test, as well as chest high‐resolution computed tomography and echocardiography with estimation of Pulmonary Artery Systolic Pressure (PASP) performed within 6 months.

The 6MWTs were performed along a hospital corridor by a qualified, experienced professional delivering standardised verbal phrases of encouragement, following current recommendations [30], except that the length of the walking circuit was reduced to 20 m to allow for the experimental setting. The oxygen flow required to prevent SpO_2_ from dropping below 90% during the 6MWT was determined on the same day and by the same operator assisting the baseline 6MWT, as part of the patient's routine ambulatory oxygen assessment, according to a published procedure [31], utilising liquid oxygen through a nasal cannula from a portable stroller, the device most commonly used in clinical practice in Italy for ambulatory oxygen [32] (see Supporting Information).

### 2.1 Experimental 6‐Minute Walking Tests

6MWTs with oxygen and medical air were conducted in sequence, according to a double‐blind randomised crossover design as previously reported [16], within 3–24 h from the baseline assessment. Medical air or oxygen, at the same flow as determined in the baseline evaluation, were delivered through nasal prongs via a 10‐m‐long plastic tubing terminating with a nasal cannula, connected to a wall outlet situated in a room midway along the corridor, allowing a walking circuit of 20 m. The same operator assisted each patient during both walking tests. The outlets (oxygen or medical air) were managed by an operator different from the one assisting with the 6MWT, according to a simple randomisation list that was computer‐generated at the start of the study by one of the authors not involved in conducting the tests. The randomisation order was stored in numbered, sealed envelopes, which were sequentially opened only at the time of the test. No other individual was allowed into the room, and the outlets and the operator were located behind a door, not visible from the corridor. After a rest period of at least 30 min on a chair, the patient was provided with an MIROxi oximeter (MIR, Rome, Italy), equipped with software dedicated to recording 6MWT results, and was connected to the tubing delivering air or oxygen, 1 min before starting the 6MWT. The oximeter was placed inside a small bag so that neither the patient nor the assisting operator could see the display during the test. As a safety measure, the oximeter's acoustic alarm was set to a limit of 70%, allowing the test to be interrupted in the event of severe desaturation, and to administer open‐label oxygen under unblinded monitoring. The patient was then asked to rest sitting in room air for at least 30 min, until comfortable, before repeating the procedure with the alternative gas mixture. At the end of each 6MWT, patients were asked to mark on a 10 cm visual analogue scale (VAS) the level of perceived dyspnoea, leg fatigue, and the sensations/feelings experienced while walking with the experimental treatment, expressed as a preference compared to walking in ambient air. After the second walk, the patients, still under blinded conditions, were also asked to mark on a similar VAS their preference between the first and the second experimental walk.

### 2.2 Statistical Analysis

All patients were analysed in the group in which they had been randomised. A generalised linear model using a Gaussian family and identity link and including order of treatment as a fixed effect [33], was employed to evaluate the effect of treatment on walking distance, heart frequency, and oxygen saturation (see additional note in the online supplement). When a 6MWT had to be interrupted because of excessive desaturation, the last SpO_2_ and HR data were carried over. The effect of patient characteristics (gender, age, BMI, smoking) and functional parameters was evaluated by adding the relevant variables to the model. Differences in parameters evaluated using VAS were analysed using the Koch adaptation of the Wilcoxon rank test [33], and correlations using Spearman rank correlation. VAS for dyspnoea and fatigue were analysed on a scale from 0 (no symptoms) to 10 cm (maximum level), while VAS for preferences were analysed on a scale from +5 cm (maximum preference for the first choice) to −5 cm (maximum preference for the comparison choice), with 0 (midline) as the point of equivalence. We also computed the ratios between VAS for dyspnoea and for leg fatigue, respectively (modified by the addition of one to avoid infinity), and walk distance in Km, as recently proposed [34]. For descriptive purposes, all data are presented in the text as the mean with 95% CI in parentheses, regardless of whether the analysis was parametric or nonparametric. A *p* value of 0.05 or lower for a two‐tail distribution was considered statistically significant. The primary outcome was 6MWT distance. From data derived from previous experiments [16], we estimated that 32 patients would provide a power of 80% to detect a difference of 28 m, corresponding to the Minimum Clinically Important Difference (MCID) for the 6MWT reported in pulmonary fibrosis [35] with an alpha of 0.05. No data were available to conduct a preliminary power analysis regarding preferences. The statistician could not be blinded to treatment, as the treatment arm was clearly apparent from the data on oxygen saturation. All the analyses were performed using STATA version 18 for Windows (Statacorp, College Station, TX).

The protocol was approved by the Ethical Committee of our Institution and was registered on a public repository (ClinicalTrials.gov ID NCT02668029).

## 3 Results

### 3.1 Patient Characteristics

We enrolled 32 consecutive patients meeting required criteria over a period of 1 year. The cohort included eight patients with non‐specific interstitial pneumonia (four with connective tissue diseases), two with fibrotic sarcoidosis, one with asbestosis, and 21 with idiopathic pulmonary fibrosis. There were no current smokers.

Demographic and baseline characteristics are presented in Table 1. The group was characterised by a restrictive pattern, with resting blood gas analysis showing a mild reduction in PaO_2_ and gas exchange. No significant differences were found in any baseline parameters between the 15 patients randomised to start with placebo and the 17 patients randomised to start with oxygen. The participant flow diagram is presented in the Supporting Information.

**TABLE 1:** Patient demographic and clinical characteristics.

	Placebo first	Oxygen first	Total
*N*	15	17	32
% Male sex	53 (28–79)	71 (49–92)	63 (46–79)
Age, years	65 (61–70)	62 (57–67)	64 (60–67)
BMI	27 (25–28)	27 (25–29)	27 (25–28)
% FVC Predicted	57 (49–64)	61 (55–67)	59 (54–63)
% FEV_1_ Predicted	59 (52–67)	64 (58–71)	62 (57–67)
% FEV_1_/FVC	85 (81–89)	85 (83–88)	85 (83–87)
% TLC Predicted	65 (59–72)	71 (65–76)	68 (64–72)
% TLCO Predicted	36 (29–43)	38 (31–44)	37 (32–42)
PaO_2_, mmHg	69 (65–72)	64 (60–69)	67 (63–70)
PaCO_2_, mmHg	40 (36–43)	40 (37–43)	40 (38–42)
A‐aO_2_ gradient, mmHg	25 (20–30)	29 (24–34)	27 (24–31)
% SpO_2_	94.4 (93.7–95.1)	94.2 (93.8–94.7)	94.3 (93.9–94.7)
PASP^a^ ≥ 40 mmHg	38% (12–64)	31% (9–54)	34% (17–52)
Baseline 6MWT, m	269 (229–308)	296 (257–336)	283 (255–311)
Oxygen, flow L/min	3.1 (2.2–4.1)	3.2 (2.3–4.1)	3.2 (2.5–3.8)

*Note*: Data presented as mean with 95% CI in parenthesis. No significant differences were found according to randomization order.

^a^ Pulmonary artery systolic pressure.

### 3.2 Randomised Placebo‐Controlled 6 Minute Walking Tests

**FIGURE 1:**
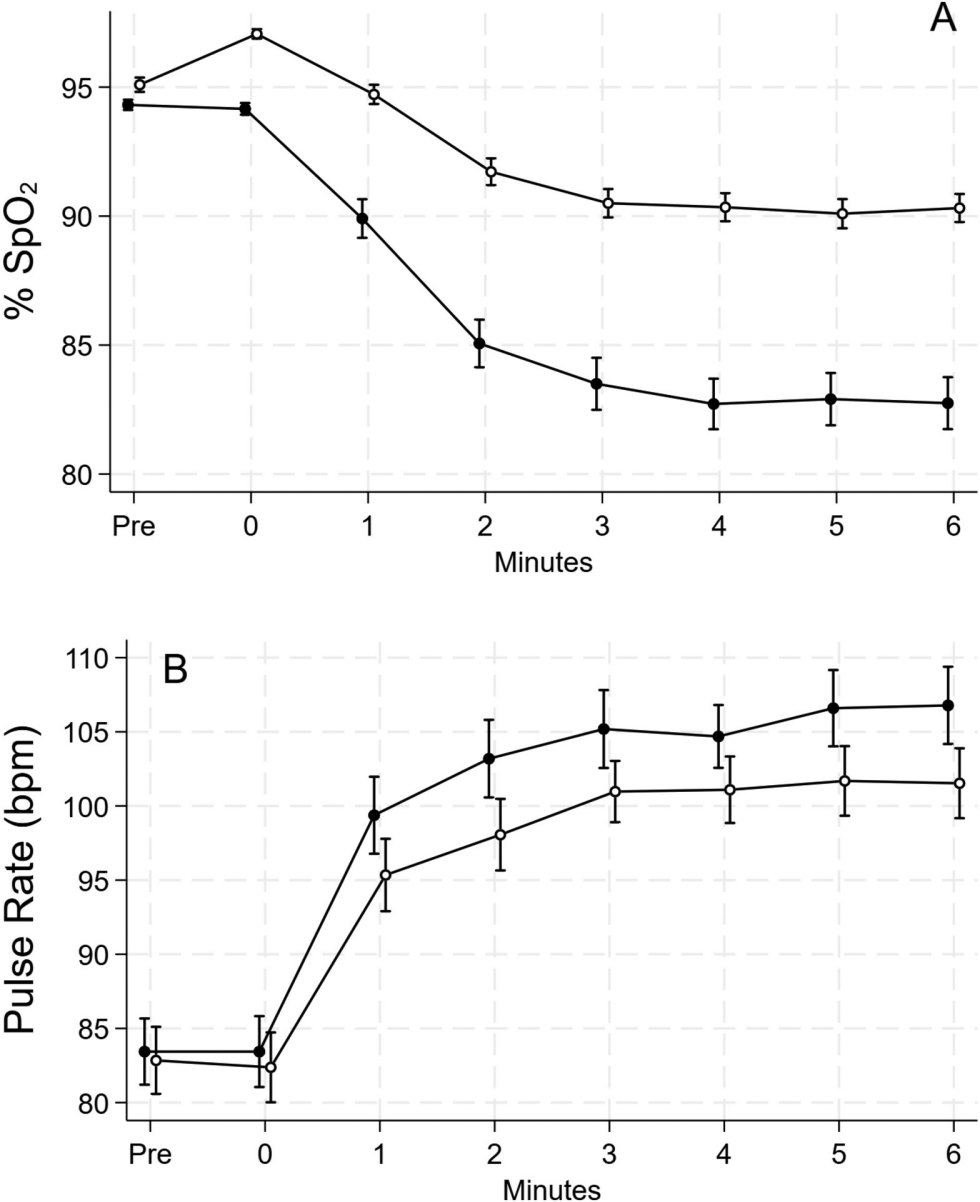
Mean values of % SpO_2_ (upper panel, A) and of pulse rate (bpm, lower panel, B) during the 6 min walking test while breathing oxygen (open circles) or placebo (solid circles). Except for baseline SpO_2_ (before wearing the nasal prongs) and, for pulse rate, time zero, the differences at all time points are all statistically significant at a *p* level of 0.003 or less. Bars represent the SE.

All patients completed the 6MWT protocol. As a preliminary assessment, we verified whether oxygen treatment prevented exertional desaturation as intended. Changes in SpO_2_ and pulse rate are shown in Figure 1. There were no differences in baseline SpO_2_: placebo 94.3% (95% CI 93.9%–94.7%), oxygen 95.1% (94.1–95.6) and in pulse rate: placebo 83.4 bpm (79.1–87.3), oxygen 82.8 bpm (78.4–87.2), before the two tests. During placebo, SpO_2_ started to decrease from the first 6MWT minute, reaching a minimum value of 81.6% (79.7–83.4). In two cases, SpO_2_ fell below 70% during the test, causing the alarm to sound and the test to be interrupted after 3 and 5 min, respectively. None of the patients had severe desaturation during oxygen, and the minimum SpO_2_ during the test was 89.6% (88.4–90.1), with a difference of 8.0% (6.5–9.5, *p* < 0.0005) between the two treatments. The highest pulse rate recorded was 109 bpm (104–114) during the placebo test and 104 bpm (100–109) during the oxygen test, with a difference of 4.0 bpm (1.0–7.1, *p* = 0.009).

**FIGURE 2:**
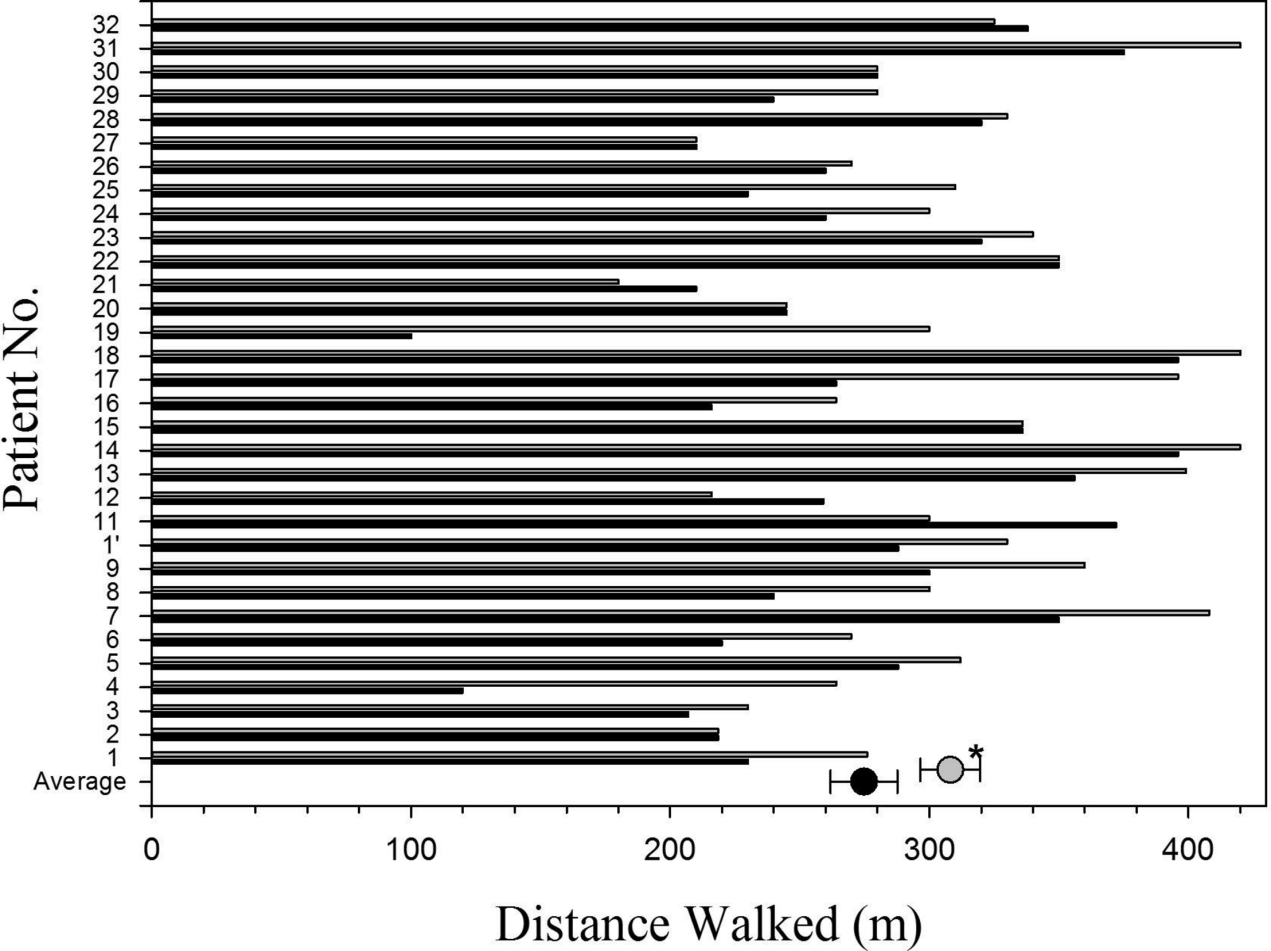
Distance walked by each patient while breathing oxygen (grey bars) or placebo (black bars). Averages are presented as circles with SE bars. *p = 0.008.

Distance walked is presented in Figure 2. The mean distance walked during placebo was 275 m (350–300), not significantly different, in a post hoc analysis, from the baseline test in ambient air, 283 m (256–310). While breathing oxygen, the distance walked was 308 m (285–331), with an increase of 37 m (10–74), after adjusting for the order of treatment (*p* = 0.008). No significant differences in the effect of oxygen on the walked distance were observed according to gender, BMI, PASP ≥ 40 mmHg, PaO_2_, PaCO_2_, or percent predicted TLC, FVC, FEV_1_, and DLCO.

The score for dyspnoea was significantly higher at the end of the test with placebo, 4.9 (4.0–5.8) compared to oxygen, 3.6 (2.7–4.4), *p* = 0.01. Similarly, the muscle fatigue score was higher for the placebo test, 4.0 (3.0–5.0) than for the oxygen test, 3.3 (2.3–4.2), *p* = 0.04 (Figure S1). Compared to placebo, 31 of the 32 patients either walked a longer distance, reported less breathlessness at the end of the test, or both when performing the test on ambulatory oxygen. The geometric mean of the modified dyspnoea to distance ratio was 19.6 (15.5–24.8) with placebo and 12.8 (10.1–16.2) with oxygen (*p* < 0.0005), and that of the modified fatigue to distance ratio was 15.2 (11.3–20.6) and 11.3 (8.7–14.4), respectively (*p* = 0.0002).

### 3.3 Preferences

**FIGURE 3:**
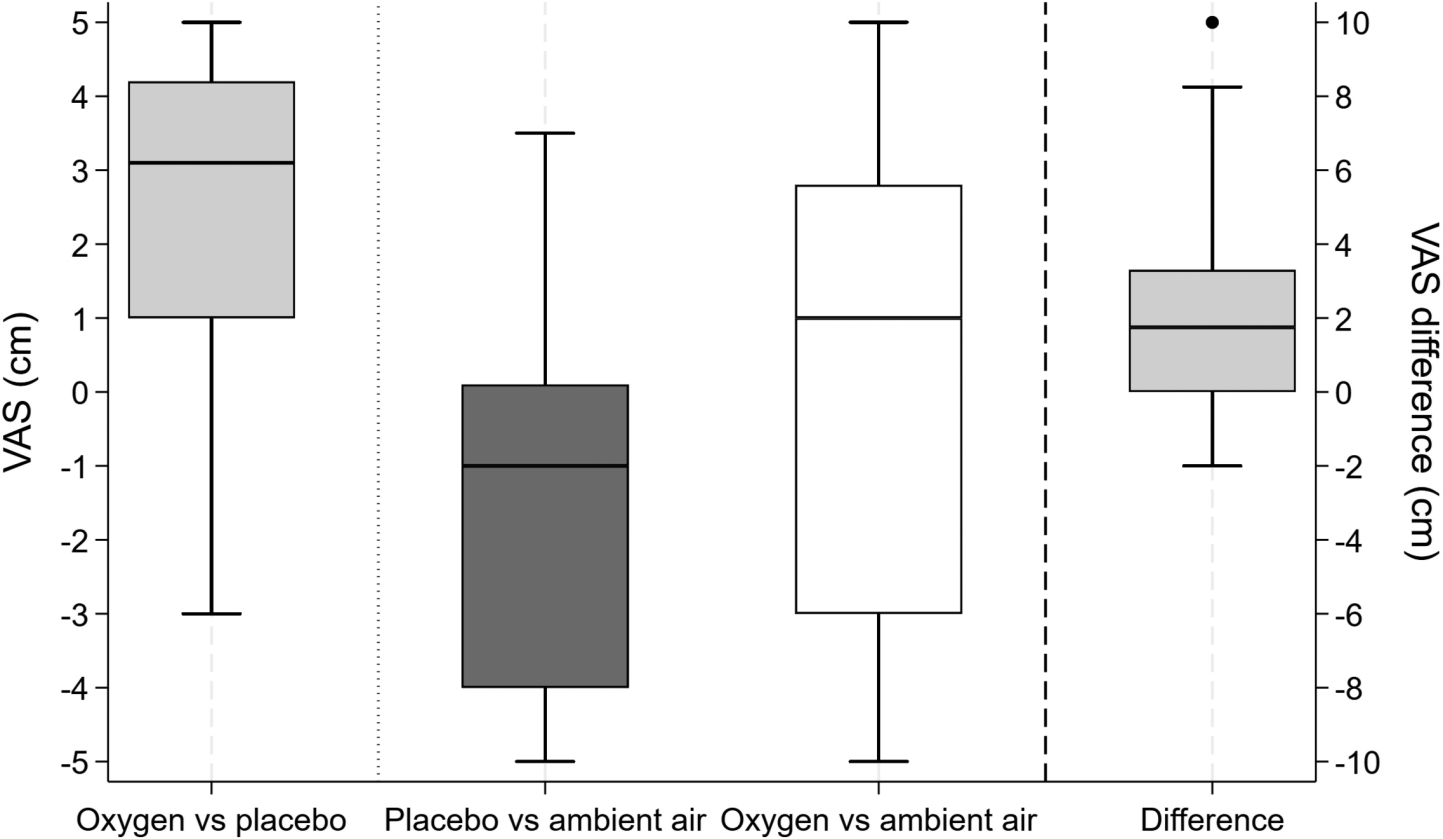
Box plots of the preferences expressed by patients for performing the walk while breathing oxygen rather than placebo (light grey), placebo rather than ambient air (dark grey), oxygen rather than ambient air (white), and the difference between the latter two (light grey, plotted on the right axis).

The results of the VAS regarding preferences are presented in Figure 3. The direct preference score between oxygen and placebo was 2.6 (95% CI 1.9–3.2) in favour of oxygen and was significantly higher than the level of equivalence (*p* < 0.0005). The VAS for walking with placebo over ambient air was −1.5 (−2.4 to −0.6), significantly lower than the level of equivalence between the two choices (*p* = 0.005), while the VAS for oxygen over ambient air was 0.4 (−0.7 to 1.5), not significantly different from the level of equivalence. Nevertheless, more than 50% of the patients preferred oxygen over ambient air. The difference between these two VAS was 1.9 (1.1 to 2.7), *p* = 0.0001, and correlated significantly with the differences between the two treatments in walked distance (rho = 0.46, *p* = 0.01), with the minimum value of SpO_2_ (rho = 0.30, *p* = 0.03), with dyspnoea at the end of the test (rho = −0.42, *p* = 0.02), and with the direct preference score between oxygen and placebo (rho 0.46, *p* = 0.01).

No significant effect of the order of treatment was detected in any of the analyses (Table S1).

## 4 Discussion

Our results confirm previous observations from our group [3, 16] and other researchers [17], that preventing oxygen desaturation during a 6MWT in patients with F‐ILD improves exercise endurance, increases walked distance by more than the MCID for the 6MWT [36], decreases dyspnoea and fatigue, and slightly reduces tachycardia, and that these effects are not due to a placebo or other non‐specific mechanism. Strengths of the study, compared to previous ones, include the careful determination of the individual oxygen flow needed to correct exercise‐induced hypoxaemia, double‐blind procedures (two key issues raised in the systematic reviews that have critically appraised the available evidence [25, 26]), and the concordance of the results of objective and patient‐reported outcomes.

We show that symptom scores were better on ambulatory oxygen than on medical air and that patients preferred ambient air over medical air, the latter administered at the same flow rate as ambulatory oxygen. This suggests that the hypothesis that breathlessness is affected by the flow, rather than by the composition of the delivered gas mixture, as previously suggested in studies conducted in static conditions, where ambulatory oxygen was not used [37], does not apply to the use of ambulatory oxygen in F‐ILD patients desaturating on exertion. We have already demonstrated that, in these patients, neither medical air nor oxygen modifies the respiratory pattern at rest, while ambulatory oxygen significantly reduces the increase in respiratory effort required during exercise [16]. These observations also corroborate the hypothesis that the positive effects on health‐related quality of life observed in the AmbOx study [18], associated with patient‐reported reduced breathlessness on exertion and increased walking ability, were not due to a placebo effect. Finally, a training effect of repeating a number of 6MWTs within a short period could be postulated. However, in a post hoc analysis, there was no difference in the 6MWT distance between baseline and placebo air, suggesting that any training effect, if present, was marginal. Furthermore, any confounding by potential learning effects on the experimental tests would in any case be dealt with by the randomization and by the adjustment for the order of treatment in the analysis (Table S1).

Notably, while the average increase in walked distance was superior to the MCID, the individual responses to oxygen in terms of distance walked and end‐test breathlessness were somewhat heterogeneous. Indeed, exercise limitation can be variously detected by the outcome measures of distance walked and breathlessness [34], which can be influenced by several other factors, such as changes in attitude, commitment, or stamina. Patients may differ in how they respond to the gas mixture, with some opting not to increase their walking rate even if breathlessness and fatigue are improved, or conversely opting to increase their effort if they feel more breathless. Nevertheless, with oxygen treatment, 91% (29/32) of patients had a lower modified dyspnoea to distance ratio with oxygen than with placebo.

These findings help to resolve residual doubts and improve the grade of evidence regarding the effectiveness of ambulatory oxygen in reducing exercise limitation in these patients [9, 18, 22], while supporting the need for studies evaluating the impact of ambulatory oxygen in everyday life in the longer term.

A potential limitation of our study is the use of a tube connected to a fixed source to administer oxygen/placebo air, a method with limited potential applications in clinical practice, where, to provide freedom of movement, the individual is usually required to carry the oxygen device (gas canister, concentrator or liquid oxygen) with a shoulder stroller, backpack, or trolley, although in the home, patients often use a long oxygen lead connected to a concentrator, without needing to carry a device during activities within the home. Our aim was primarily to fill a gap in the available evidence regarding a possible placebo effect of ambulatory oxygen in the 6MWT, and we do not propose our setting as a test to be used in clinical practice. We chose a light delivery system of the gas mixture because we were mostly interested in the comparison between oxygen and placebo, minimising the interference of the method of delivery, and because its level of inconvenience was reasonably comparable (though not equal) to that of a lightweight stroller with liquid oxygen, the most frequently used device for delivering ambulatory oxygen in Italy [32]. The negative impact of using different delivery systems could vary [38, 39], and more research is needed on the effectiveness and acceptability of different devices [23, 38, 39, 40]. Thus, our data suggest the need to systematically test for exercise desaturation all F‐ILD patients with exertional breathlessness, considering the ability of ambulatory oxygen to improve exercise tolerance for those with exercise‐induced desaturation, regardless of any other potential, yet unproven, long‐term benefits. In clinical practice, however, the choice will require individual testing using the same kind of device which would be available at home, ideally extending the evaluation to the patient's living environment [18, 24].

Our protocol also measured the global perceived effect (preference between walk with oxygen and ambient air) and the possible placebo effect of the treatment devoid of oxygen's specific effect (preference between placebo and ambient air). In the comparison between medical and ambient air, most patients expressed a preference for ambient air. As a consequence, the preference between oxygen and ambient air, resulting from a mixture of the positive effect of oxygen and the perceived downsides of the treatment, provides heterogeneous results, with only slightly more than 50% of patients expressing a preference for oxygen.

Several studies have shown that ambulatory oxygen tends to be underused by patients [32, 41, 42, 43, 44]. Factors contributing to underuse include practical issues (weight, bulk, costs, care, refilling), inconvenience (hampering, fear of loss or breakage, accidents, running out of oxygen), and psychological factors (stigma, self‐image, unpreparedness, prejudices about possible addiction, poor acceptance of the disease, especially in the early stages of adaptation to the disease) [18, 27, 45, 46, 47], and might differ between patients with isolated exertional hypoxia and those already on long‐term oxygen therapy for resting hypoxia [27].

While preferences have been previously used to compare the acceptability of different devices [23], to our knowledge this is the first study to compare patient preferences regarding ambulatory oxygen versus placebo, recorded at the time of the 6MWT. The disparity between the clear objective benefit provided by oxygen compared to placebo air on the one hand, and the more heterogeneous preferences reported by patients for oxygen compared to ambient air on the other, suggests the existence of a negative preconception likely related to individual concerns, attitudes, and beliefs, which could affect the degree with which treatment with ambulatory oxygen is embraced. The previously mentioned limitation of our study design relative to the oxygen delivery method actually turns into an advantage when interpreting the data regarding patient preferences. Indeed, in this case, the negative attitudes towards treatment may be mostly influenced by psychological factors and largely independent from the bulk or weight of the oxygen delivery method, considering the very low weight method of delivering oxygen or medical air in this study. Although our study does not provide any further evidence, this suggests that the psychological factors mentioned above [18, 27, 45, 46, 47] not only can impact the willingness to use ambulatory oxygen but might also interfere directly with the individual perception of its effects. Investigating this hypothesis would require further studies; nevertheless, more than half of patients gave a higher preference score to oxygen compared to ambient air.

Since the 6MWT is often performed before prescribing ambulatory oxygen, our results suggest that asking for preferences between the test without oxygen and the one with ambulatory oxygen delivered with the device to be used in practice could help highlight and address these issues. Further studies are needed to clarify if and how the preferences expressed after the 6MWT predict subsequent use and benefit from ambulatory oxygen, whether the negative preferences expressed by a proportion of patients towards ambulatory oxygen in this study are related to the same factors identified in relation to underuse in patients already on treatment with oxygen, how they are related to methods of delivery, and whether they can be modified. Investigating and addressing these issues requires a personalised approach within a context of shared decision‐making, integrating the patient's values, experiences, concerns, preferences, and expectations with available evidence and resources in a respectful and compassionate clinical relationship to empower the patient to choose if and when to use ambulatory oxygen [29, 48, 49, 50]. Thus, our data support the approach of recent guidelines towards shared decision making [21] and suggest that inviting patients to express their preferences after experiencing ambulatory oxygen during the 6MWT and incorporating this into the clinical discussion could improve patient involvement, strengthen the therapeutic relationship, empower the patient, and lead to a more informed and personal choice regarding the prescription of ambulatory oxygen for exertional breathlessness in patients with F‐ILD.

## Author Contributions

**Giuseppina Ciarleglio:** conceptualization (equal), investigation (equal), writing – original draft (equal), writing – review and editing (equal). **Paolo Cameli:** conceptualization (equal), writing – review and editing (equal). **David Bennett:** conceptualization (equal), writing – review and editing (equal). **Behar Cekorja:** conceptualization (equal), investigation (equal), writing – review and editing (equal). **Paola Rottoli:** conceptualization (equal), supervision (equal), writing – review and editing (equal). **Elisabetta A. Renzoni:** conceptualization (equal), methodology (equal), writing – original draft (equal), writing – review and editing (equal). **Piersante Sestini:** conceptualization (equal), data curation (equal), formal analysis (equal), methodology (equal), project administration (equal), supervision (equal), writing – original draft (equal), writing – review and editing (equal). **Elena Bargagli:** conceptualization (equal), supervision (equal), writing – review and editing (equal).

## Ethics Statement

The protocol was approved by the Ethical Committee of our Institution and was registered on a public repository (ClinicalTrials.gov ID NCT02668029).

## Conflicts of Interest

This study was conducted as independent academic research at the Respiratory Diseases Unit of the University of Siena, without external sponsors. The authors declare no conflicts of interest. Elisabetta A. Renzoni is an Editorial Board member of Respirology and a co‐author of this article. She was excluded from all editorial decision making related to the acceptance of this article for publication.

## Supporting information


**Data S1.** Supporting Information.

## Data Availability

Anonymized data are available in digital form from the corresponding author upon reasonable request for legitimate scientific purposes.
