# Supplementary material for: Objective Effects and Patient Preferences for Ambulatory Oxygen in Fibrotic Interstitial Lung Disease With Isolated Exertional Hypoxaemia: A Placebo‐Controlled 6‐Minute Walk Test Study
Source: Respirology. 2025 Mar 11;30(7):644–51. doi: 10.1111/resp.70020 (PMC12231771; doi:10.1111/resp.70020)
Supplement: Supplementary file 1 — Data S1. Supporting Information. [file RESP-30-644-s001.pdf]

Objective Effects and Patient Preferences for Ambulatory Oxygen in Fibrotic Interstitial Lung Disease with isolated exertional hypoxemia: A Placebo-Controlled 6-Minute Walking Test Study

Ciarleglio G, Cameli P, Bennett D, Cekoria B, Rottoli P,
Renzoni EA, Sestini P, Bargagli E

Supplementary file

Study Participant Flow Diagram

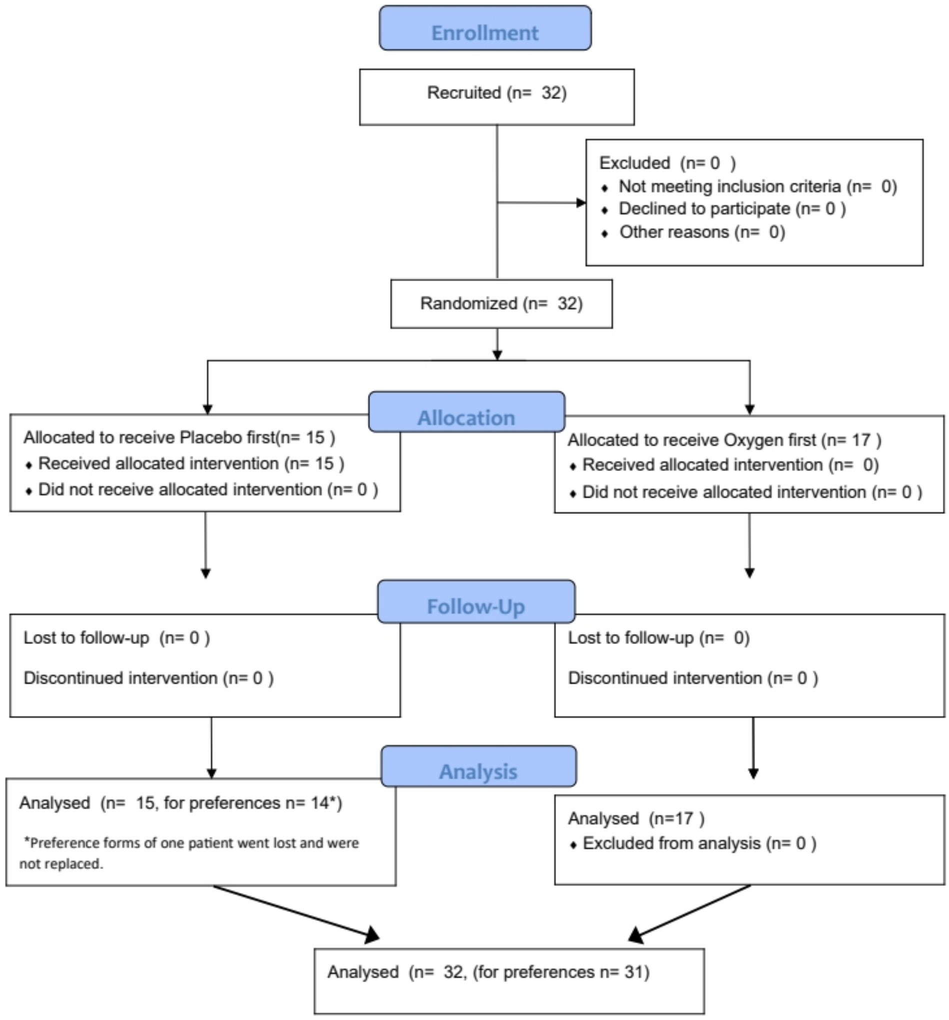

Hospital Test for Exercise Oxygen Prescription[1]**.**

A conventional 6MWT was conducted by an experienced operator as part of the routine clinical assessment, in the same or a similar corridor as the experimental ones. Oxygen saturation was monitored using a portable fingertip pulse oxymeter that was checked and recorded manually by the operator at each pass. Only oxygen saturation and walked distance were recorded. After completing the conventional 6MWT in ambient air, patients whose oxygen saturation dropped below 89% underwent further tests to determine the oxygen flow necessary to prevent desaturation. These tests utilized liquid oxygen delivered via a nasal cannula connected to a portable stroller, the device most commonly used in clinical practice in Italy for ambulatory oxygen. The patients carried both the oxygen stroller and a finger oximeter. If the minimum SpO2 during the previous test ranged from 85% to 89%, the flow was increased by 1 L/min; if it was below 85%, the flow was increased by 2 L/min. Patients rested for at least 20 minutes between tests. The operator checked SpO2 during each pass, and if it dropped below 89%, the test was interrupted and repeated after sufficient rest, with the oxygen flow increased according to the same scheme as before. Oxygen flow was deemed adequate if SpO2 remained >89% throughout the test. Patients were enrolled in the study only after the appropriate flow was determined. All eligible patients agreed to participate.

[1] Guyatt GH et al, Am J Respir Crit Care Med 163:942-946, 2001

Additional note on statistical analysis In a two-period crossover design, it is intrinsically impossible to assess carryover effects, and the validity of the results relies on the reasonable assumption that no carryover has occurred[2]. To address this, we ensured washout periods of at least 30 minutes, as there is no clinical or biological evidence suggesting that the effects of supplementary oxygen in these patients persist for more than a few minutes. Nevertheless, if any carryover effect of oxygen administration were to occur, it would result in a prolongation of its effect during the subsequent period with placebo, leading to an underestimation of the true efficacy of ambulatory oxygen.

Supplementary table 1.

	Order of treatment	
	Placebo 1st	*Oxygen 1st*
Min % SPO2	8 (6–9)	8 (6–11)
Max HR (bpm)	-4 (-6–-2)	-5 (-9–-2)
6MWT distance (m)	37 (11–63)	30 (4–57)
Dyspnea (cm)	-1.1 (-1.9–-0.2)	-1.5 (-2.5–-0.5)
Fatigue (cm)	-0.9 (-1.8–0)	-0.6 (-1.7–0.4)
Dyspnea/distance	-7.3 (-11.3– -3.3)	-10.3 (-20.1– -0.5)
Fatigue/distance	-6.3 (-10.6– -2.0)	-8.1 (-17.2– 0.9)
Preferences: Oxygen vs Placebo	2 (0.9–3.1)	3.2 (2.4–3.9)
Placebo vs AA	-1.5 (-2.4–-0.6)	-1.8 (-3.2–-0.5)
Oxygen vs AA	0.4 (-1.3–2.1)	0.1 (-1.5–1.6)
Difference	1.5 (0.4–2.5)	2.3 (1–3.5)

Supplementary Table 1: Differences in relevant outcomes (Oxygen - Placebo), according to order of treatment. M with 95% CI in parenthesis. None of the comparisons between the two treatment orders reached statistical significance

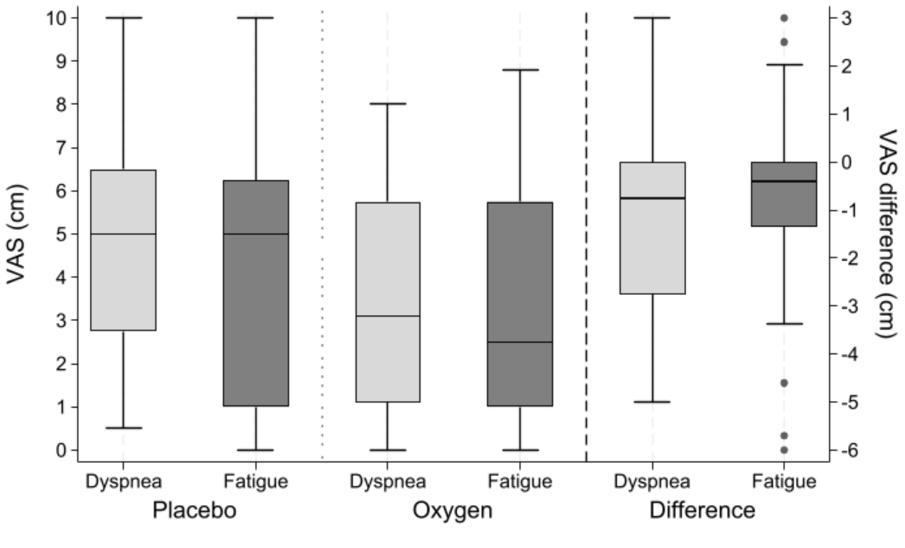

Supplementary Figure 1: Box plot of scores for dyspnea (light gray) and fatigue (dark gray) at the end of the 6MWT with placebo (medical air) or oxygen, and of their differences between oxygen and placebo (plotted on the right axis). Both scores were significantly lower after oxygen than after placebo (P=0.01 and 0.04, respectively, Wilcoxon signed rank test)

[2]Senn S, Stat Methods Med Res,1994 3:303-324
